# Immobilization of Pt nanoparticles on hydrolyzed polyacrylonitrile-based nanofiber paper

**DOI:** 10.1038/s41598-021-90536-5

**Published:** 2021-06-01

**Authors:** Soon Yeol Kwon, EunJu Ra, Dong Geon Jung, Seong Ho Kong

**Affiliations:** 1 School of Electronic and Electrical Engineering, Kyungpook National University, Daegu, 41566 Republic of Korea; 2 Fineroute academy, Hwaseong, Gyunggi-do 18505 Republic of Korea; 3 Safety System R&D Group, Korea Institute of Industrial Technology (KITECH), 320, Techno sunhwan-ro, Goryeong-gun, 42994 Republic of Korea

**Keywords:** Chemistry, Engineering, Materials science, Nanoscience and technology

## Abstract

The electrochemical activity of catalysts strongly depends on the uniform distribution of monodisperse Pt nanoparticles without aggregates. Here, we propose a new hydrolysis-assisted smearing method for Pt loading on a free-standing paper-type electrode. Polyacrylonitrile (PAN)-based nanofiber paper was used as the electrode, and it acted as a Pt support. Hydrolysis of the electrode tripled the number of active nucleation sites for Pt adsorption on the PAN nanofibers, thereby significantly enhancing the wettability of the nanofibers. This facilitated the uniform distribution of Pt nanoparticles without aggregate formation up to 40 wt% (about 0.8 mg/cm^2^) with a particle size of about 3 nm. The catalytic current of the hydrolyzed Pt electrode in CH_3_OH/H_2_SO_4_ solution exceeded 213 mA/cm^2^ Pt mg, which was considerably greater than the current was 148 mA/cm^2^ Pt mg for an unhydrolyzed electrode.

## Introduction

The catalytic efficiency of Pt nanoparticles for hydrogen or methanol decomposition is governed by the uniformity of the nanoparticle distribution, the monodisperse size of the nanoparticles, the presence of aggregation, and the Pt loading content of the nanoparticles, all of which strongly depend on the choice of electrodes and the Pt loading method. While many methods have been proposed for preparing Pt nanoparticles^1–4^, most of them are based on liquid-phase reactions. In particular, Pt nanoparticles have also been prepared through gas phase evaporation. Both approaches usually involve the aggregation of Pt nanoparticles.

Generally, carbon materials such as porous carbons^1,2^, activated carbons^5^, carbon nanotubes^4^, and chemical-vapor-deposition-grown nanofibers^6^ are used as Pt supports. After Pt particles are loaded onto these materials, they are further processed to fabricate an electrode on the diffusion layer. A key requirement for highly efficient catalytic activity is a uniform distribution of monodisperse Pt nanoparticles without any aggregate on a robustly formed conductive electrode^7,8^. The formation of carbon electrodes from powder is rather intricate and involves the use of binders and ionomers. The use of the former is a drawback since binders have high resistance. Therefore, if possible, the use of free-standing paper-type electrodes is desirable for Pt loading.

Electrospinning of polymers is a very useful method for preparing fibers with diameters in the sub micrometre to nanometre range^9,10,11^, and the fibers can be in the form of free-standing yarn^12^, aligned fibrous arrays^13^, or paper^14^. In particular, electrospinning of polyacrylonitrile (PAN) and the subsequent stabilization and carbonization of the fiber prepared yield carbon nanofibers^15^. The use of PAN as a precursor is advantageous for obtaining robust nanofibers, which can be used as a free-standing carbon paper electrode for energy storage devices without requiring a binder, unlike the case of activated carbons^16^.

Here, we propose a new hydrolysis-assisted smearing (HAS) method for Pt loading on a binder-free electrode. HAS method can uniformly distribute Pt nanoparticles on the surface of nanofibers by increasing an electrostatic attraction between a Pt precursor and an Electrospun PAN nanofiber paper used as an electrode^15^. In order to make the number of active nucleation sites for Pt adsorption, it was hydrolyzed through a chemical reaction on the surface of PAN nanofiber using KOH solution. Pt(acac)_2_ was used as Pt precursor, and it is easily transformed to Pt nanoparticles by heat treatment below 1000 ℃. Uniform distribution of Pt loading was successfully produced without aggregate formation up to 40 wt% (0.8 mg/cm^2^) with a particle size less than 3 nm using HAS method. The ratio of the forward oxidation peak intensity to the backward oxidation peak intensity was 1.83, similar to that for Pt–Ru-loaded carbon black.

## Experimental

### Experimental procedure for preparing electrospun PAN nanofiber paper

PAN and *N*,*N*-dimethylformamide (DMF) were purchased from Aldrich Chemical. PAN was dissolved in DMF solution with a concentration of 10 wt%, and the resulting polymer solution was used for electrospinning with a variable high-voltage power supply (maximum dc voltage of 35 kV). The bias voltage was optimised and fixed at 20 kV, and the distance between the needle and the collector was 15 cm. A metal drum collector with a diameter of 15 cm, wrapped in aluminium foil, and rotating at 1000 rpm was used for collecting the electrospun nanofibers. The electrospun nanofiber paper was stabilised at 280 °C at a ramping rate of 1 °C/min for one hour in air, following which its colour changed from white to dark brown.

### Pt loading on PAN nanofiber paper

The PAN nanofiber paper was hydrolysed in 0.1 M KOH solution for two hours at various temperatures. The remaining potassium was removed by first washing with 3 M HCl solution and then with deionised water. Thereafter, the paper was dried in an oven at room temperature overnight. Pt solution was prepared by dissolving Pt(acac)_2_ in acetone, and the hydrolysed PAN nanofiber paper was immediately soaked in the spraying Pt solution. The Pt loading content was determined from the volume of prepared Pt solution. Finally, the Pt-loaded nanofiber paper was carbonised at 800 °C under Ar atmosphere.

### Data collection

The surface morphology and sizes of Pt particles were determined using a scanning electron microscope (SEM; JSM6700F, ZEOL) and a transmission electron microscope (TEM; JEM2100F, ZEOL). The Pt loading content was obtained via thermogravimetric analysis (TGA), and chemical analysis was performed using X-ray photoelectron spectroscopy (XPS; ESCA2000, VG Microtech, England). The size of Pt particles was obtained using an X-ray diffractometer (XRD; 12 kW, Rigaku) for the 2θ range 5°–80°. The surface charge was obtained through electrophoretic light scattering by using ELS-8000 (Otsuka Electronics). Cyclic voltammetry was performed using a Solartron 1400 series three-electrode system. The samples were ground and dispersed in isopropanol by sonication, and a certain amount of the solution was then dropped onto the graphite electrode. The test was performed in a 1.0 M CH_3_OH + 0.5 M H_2_SO_4_ solution at a scanning rate of 20 mV/s. Immediately before CV measurement, the CH_3_OH/H_2_SO_4_ solution was bubbled with N_2_ gas for 30 min to remove molecular oxygen.

## Results and discussion

**Figure 1:**
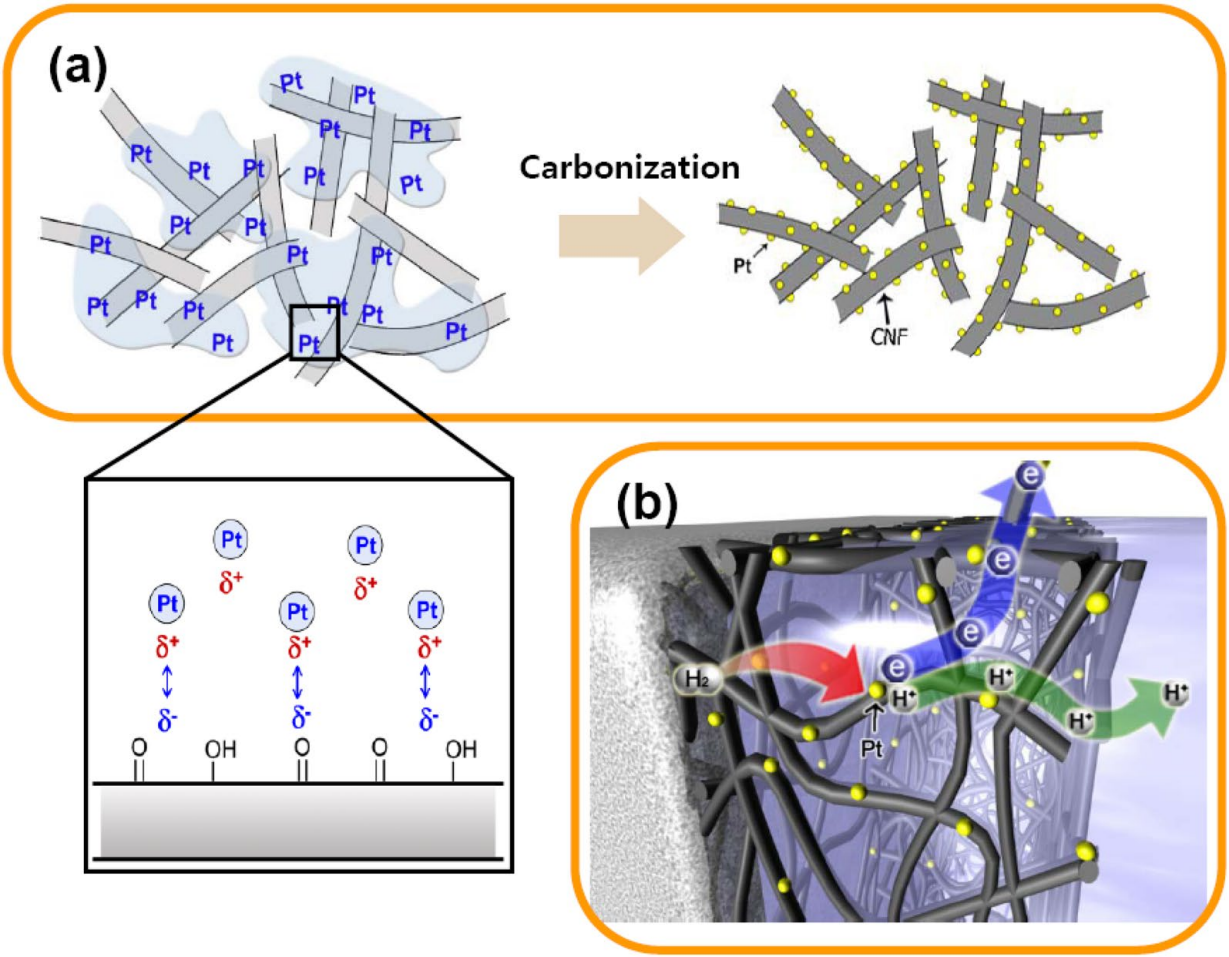
Schematic illustration of the HAS method. (**a**) Droplets of Pt(acac)_2_ solution on the hydrolysed nanofiber paper and smearing of the droplets on the network of nanostructured nanofibers. Pt was adsorbed on the negatively charged (hydrolysed) nanofiber surface. (**b**) A schematic of the Pt-loaded nanofiber network and electrolytes forming a three-phase interface. Drawn by the 3D-Max program student version.

Schematic of the HAS method as a graphical depiction of the HAS method developed in this study is presented in Fig. 1a. The fabrication of the electrospun PAN nanofiber paper has been described elsewhere^12^. The designed amount of platinum(II) acetylacetonate (Pt(acac)_2_) solution was sprayed repeatedly on the hydrolyzed nanofiber paper, as described in the Experimental Section. The Pt(acac)_2_ solution could be easily smeared on the network of nanotextured nanofibers. The presence of extra carbonyl groups that were formed in the hydrolysis process enhanced the negative charge on the nanofiber surface^17^ and acted as electrophilic sites, providing nucleation sites for Pt adsorption as discussed later^18^. Carbonization led to the formation of Pt nanoparticles, and the diffusion of Pt atoms during carbonization was limited by the strong chemical bonding between carbon and Pt^18^. The nanofiber network described herein can be used as an electron channel, as shown in Fig. 1b, in which protons are transported through Nafion to the opposite electrode. Since the carbon nanofiber paper is highly porous (porosity > 90%), the active Pt sites are directly accessible to hydrogen gas or methanol, and the Nafion electrolyte comes in direct contact with the Pt catalyst. Thus, robust three-phase contact is achieved.

### Morphology of Pt-loaded PAN-based nanofiber paper

**Figure 2:**
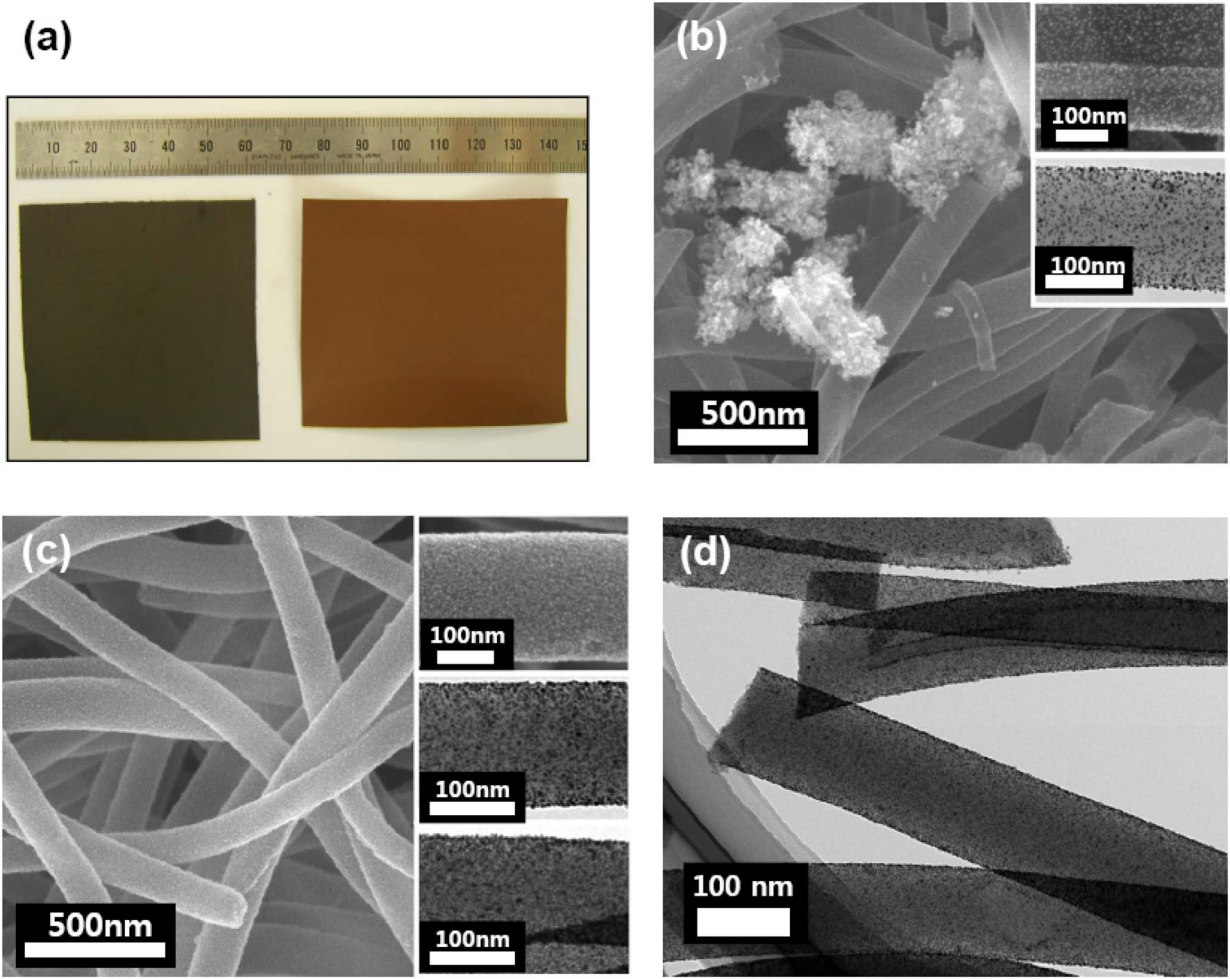
Image taken by SEM and TEM instrument (**a**) photographs of PAN nanofiber paper with 30 wt% Pt loading after stabilisation (brown) and after carbonisation at 800 °C (black). (**b**) An SEM image of unhydrolyzed Pt-loaded carbonised nanofiber with Pt aggregates (white spots); the inset shows SEM and TEM images of an individual Pt-loaded nanofiber. (**c**) An SEM image of a Pt-loaded carbonised nanofiber subjected to hydrolysis at 50 °C; SEM and TEM images for 30 wt% Pt-loaded nanofiber (top and middle panels in the inset) are also shown along with a TEM image for 40 wt% Pt-loaded nanofiber (bottom inset). (**d**) High-resolution TEM images of samples with 30 wt% Pt loading. The surface morphology were observed by using a scanning electron microscope (SEM; JSM6700F, ZEOL) and a transmission electron microscope (TEM; JEM2100F, ZEOL).

The electrospun nonwoven PAN nanofibers paper with diameters on the order of about 300 nm were prepared for use as an electrode for loading Pt nanoparticles. The conductivity of the PAN nanofiber paper was about 0.5 S/cm after carbonization at 800 °C under Ar gas^16^. Its thickness could be easily controlled up to a few hundred micrometres by varying the amount of PAN solution used. The specific surface area of the nanofiber paper determined from the N_2_ adsorption isotherm was 33 m^2^/g, and it mostly comprised external surface area^19,20^. The color changed from white to dark brown upon stabilization. The morphology of the paper and individual nanofibers remained intact during hydrolysis (right part of Fig. 2a), although the surfaces of the individual nanofibers were chemically modified. The Pt solution was prepared by dissolving Pt(acac)_2_ in acetone, and it was sprayed on the hydrolyzed PAN nanofiber paper continually. The Pt loading content was determined by the concentration and volume of the Pt solution. The Pt-loaded nanofiber paper was finally carbonized at 800 °C under Ar atmosphere (black color, left part of Fig. 2a). The paper density after carbonization was below 0.1 g/cm^3^ without Pt loading, which was considerably lower than that (2.2 g/cm^3^) of nonporous carbon, and it indicated a porosity exceeding 90%. Notably, the specific surface area after Pt loading increased to 138 m^2^/g following hydrolysis, which was ascribed to the presence of Pt nanoparticles. SEM morphologies of the carbonized Pt-loaded nanofiber paper are presented in Fig. 2b,c. For 30 wt% Pt loading by the HAS method, Pt nanoparticles were coated well on the individual nanofibers before hydrolysis (Fig. 2b). However, relatively large aggregates of Pt particles (diameter ≈ 400 nm) were often observed on the nanofibers. These large aggregates were observed more often when the loading content was increased to 40 wt%. However, the aggregates disappeared after hydrolysis at 50 °C (Fig. 2c,d). Instead, Pt nanoparticles were more uniformly and more densely packed after hydrolysis, as can be observed in the SEM images and TEM images in Figs. 2c (the top and middle insets) and 2d. The Pt particle density after hydrolysis was about 0.038 particles/nm^2^ at 40 wt%, which was thrice the density without hydrolysis. The average particle sizes were determined to be 3 ± 0.6 nm on the basis of TEM images, and the size of Pt nanoparticles did not change significantly after hydrolysis for the Pt loading content of 40 wt% (the bottom inset in Fig. 2c).

### Effect of hydrolysis temperature on Pt nanoparticle size

**Figure 3:**
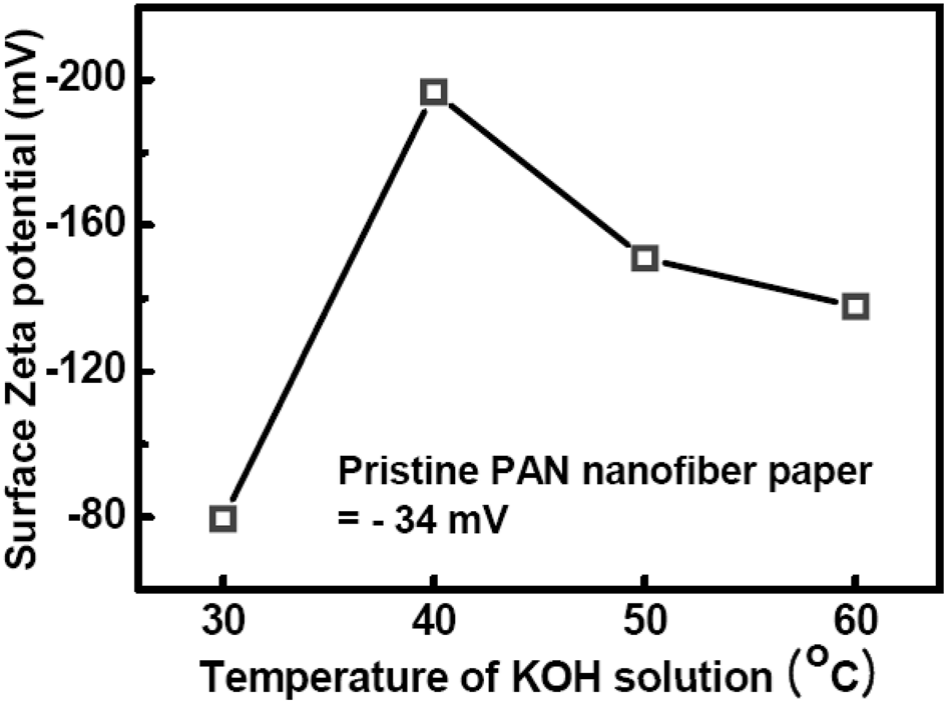
Surface zeta potential of the PAN nanofiber paper as a function of the KOH solution temperature.

The effectiveness of hydrolysis depended on the reaction temperature, reaction time, and KOH concentration^17^. By fixing the reaction time and KOH concentration, we optimized the reaction temperature. The surface charge was determined from electrophoretic light scattering measurements. The surface zeta potential of the pristine PAN nanofiber paper was measured to be − 34 mV. The highest (− 200 mV) zeta potential was obtained near 40 °C (Fig. 3), and at this zeta potential, the wettability of the electrode was maximized such that Pt droplets could be immediately soaked into the surface of the nanofibers, making the HAS method more effective.

**Figure 4:**
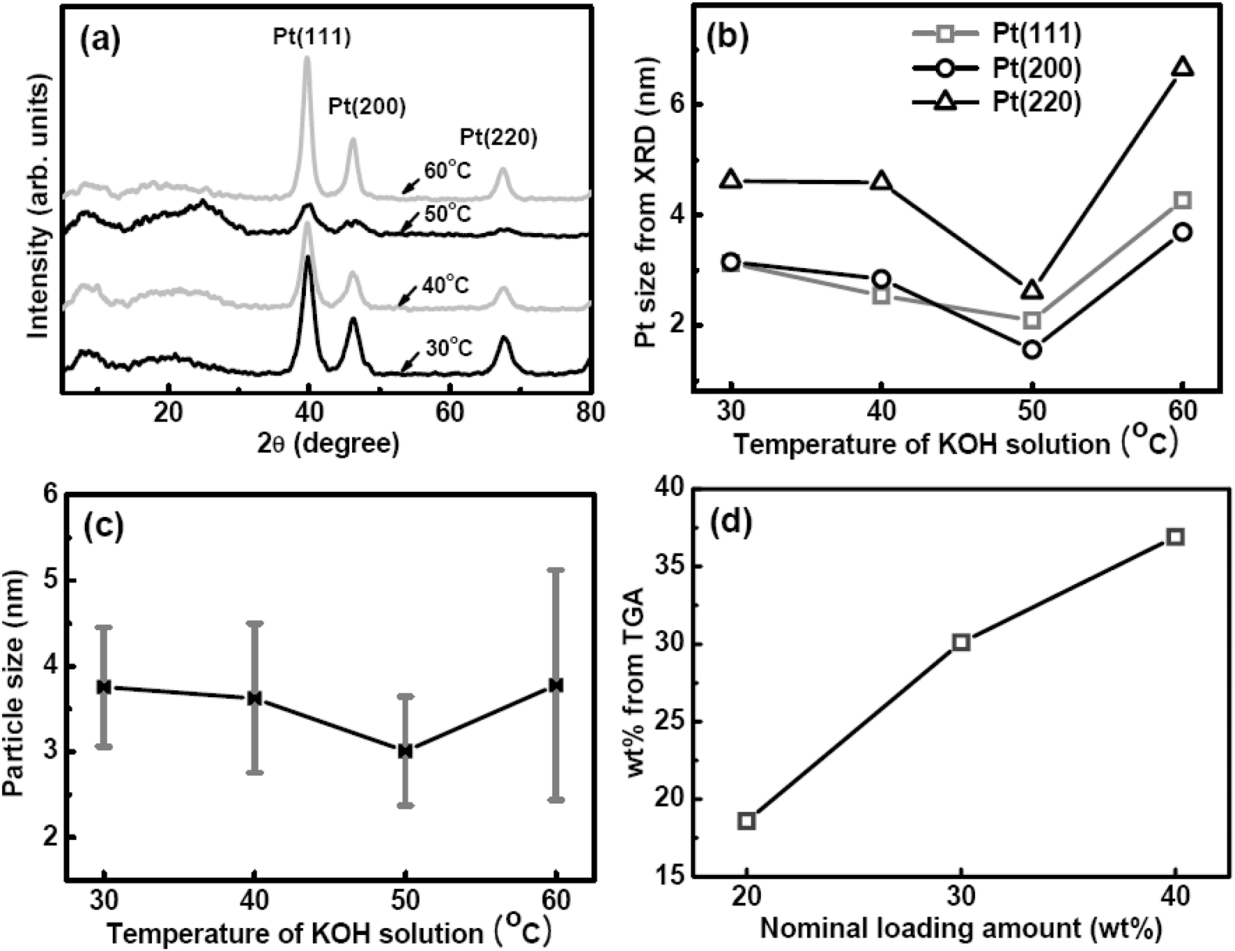
(**a**) XRD curves for different reaction temperatures employed for hydrolysis. (**b**) The sizes of Pt nanoparticles as determined from XRD peaks by using Scherrer’s formula. (**c**) The sizes of Pt nanoparticles as estimated from TEM images. (**d**) The loaded Pt content determined through TGA in terms of the nominal loading content.

Figure 4a presents the X-ray diffraction (XRD) pattern for 30 wt% Pt loading as a function of the reaction temperature. The Pt particle size was estimated from Scherrer’s formula, d (nm) = 0.9λ/βcos(2θ), where λ and β are the wavelength of the X-ray source and the full width at half-maximum of each diffraction peak corresponding to the face-centred cubic structure of Pt, respectively. The Pt particle size was the smallest at 50 °C for all the diffraction peaks, although the absolute average size differed for the different peaks (Fig. 4b). A similar variation of the particle size with the reaction temperature was observed for the sizes of the Pt particles obtained from TEM images (Fig. 4c). The best conditions were achieved at 50 °C, at which the average particle size was 3 nm with the minimum standard deviation. At the higher reaction temperature of 60 °C, the Pt particles started aggregating and the particle size range increased, with particle sizes often reaching nearly 7 nm. The Pt loading amount was obtained through TGA. The nominal value of the initially designed Pt content determined from the volume of the Pt solution was in excellent agreement with the Pt content measured using TGA, particularly for low Pt content (Fig. 4d). This is another advantage of the HAS method.

### Analysis of surface chemistry by XPS

**Figure 5:**
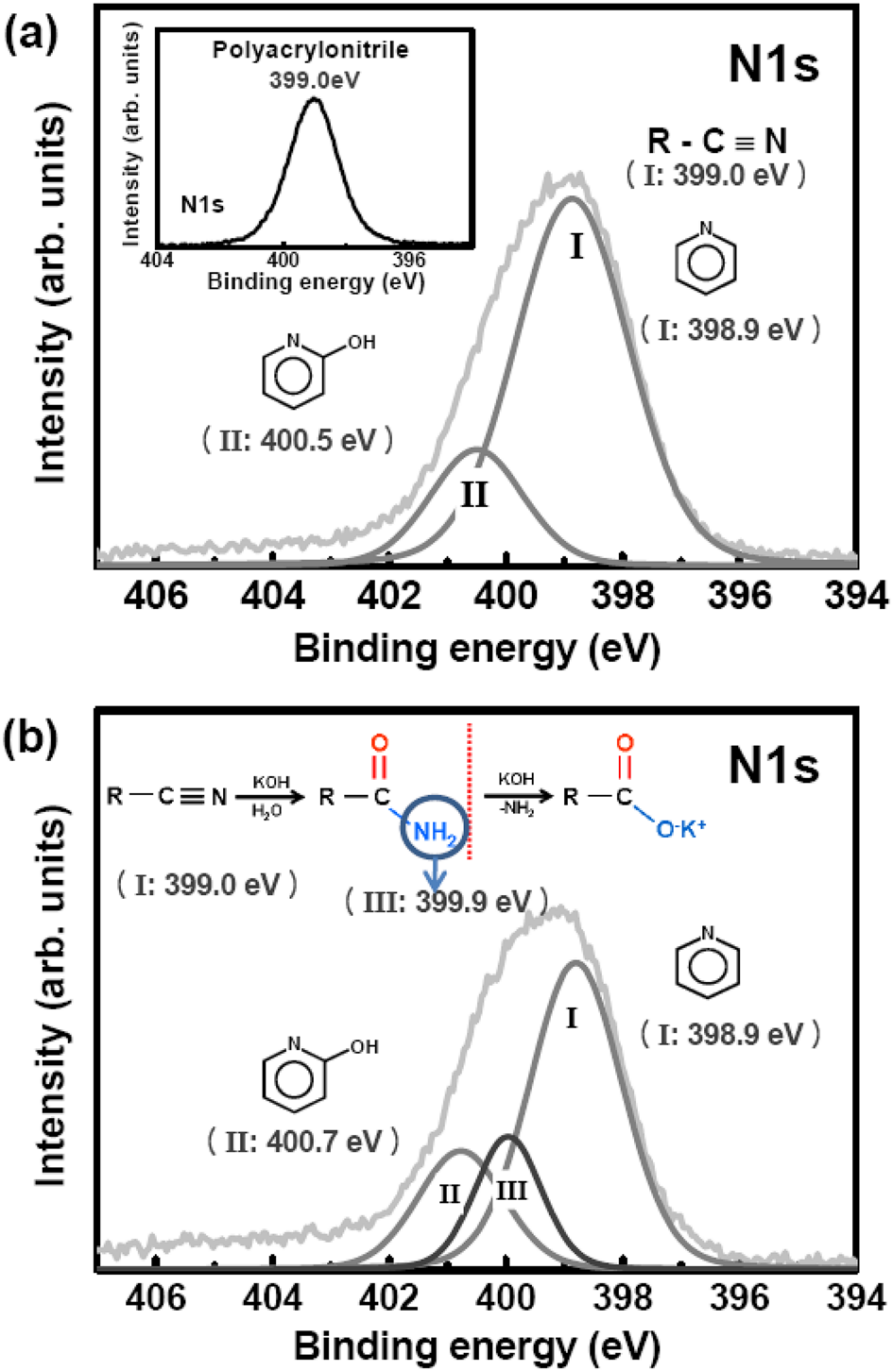
N1s peaks for the (**a**) pristine and (**b**) hydrolysed PAN nanofibers.

**Figure 6:**
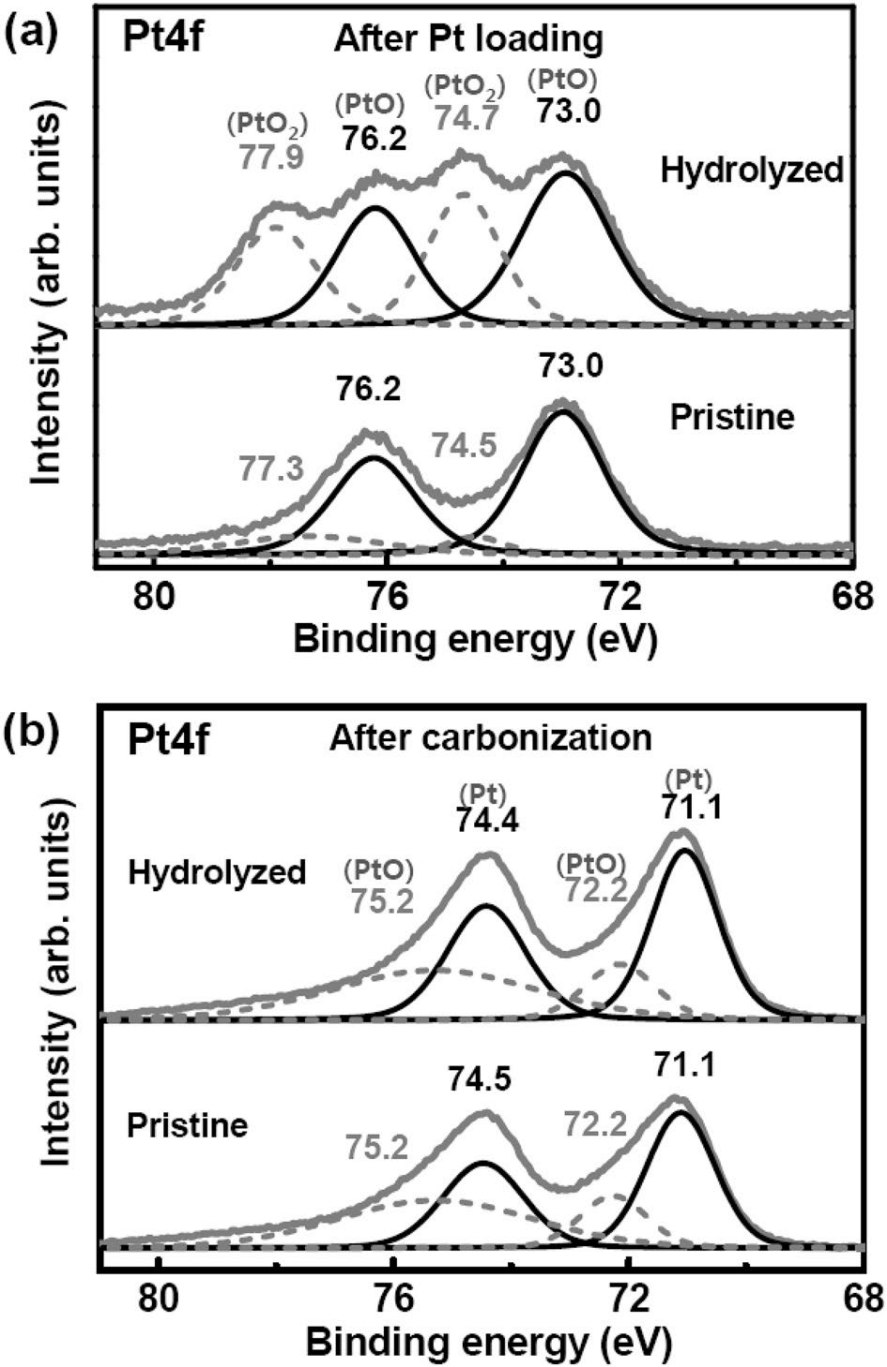
Pt4f. peaks for the pristine and hydrolysed PAN-based nanofibers (**a**) after Pt loading and (**b**) after carbonisation.

In order to clarify the adsorption mechanism of Pt nanoparticles on hydrolyzed PAN nanofibers, we provide XPS analysis data in Figs. 5 and 6. PAN comprises a polyacryl backbone with side-chain nitriles. A pristine sample without Pt loading showed an N1s peak near 399.0 eV (inset of Fig. 5a), which is attributed to a nitrile group^21^. A cyclisation reaction occurred during stabilization, resulting in functional groups such as C-N conjugations, carbonyl groups, and hydroxyl groups being formed; however, some C≡N bonds remained unchanged^22,23^. After stabilization without Pt loading, the N1s peak showed a rather broad tail near the high-energy side, which was deconvoluted into two peaks (Fig. 5a). The main groups were the nitrile group that remained, a newly formed pyridine group near 398.9 eV (I), indistinguishable peaks, and small pyridone groups near 400.5 eV (II)^24^.

The carbon atom in the CN group is nucleophilic and therefore attracts OH − ions during hydrolysis. Consequently, the nitrogen atom is converted into hydroxyl imine (NH), which is further transformed into NH_2_ upon accepting H ions from the OH group in a tautomerization process^25^. This process involves the incorporation of oxygen atoms, as observed in our case. The reaction can be driven further by incorporating more oxygen atoms to form negatively charged carboxylic acid, which requires a high reaction temperature of about 200 °C, as shown in the flow chart of Fig. 5b^24^. Since our reaction temperature was maintained at a moderate value of 30–60 °C, the this reaction was unlikely to occur. The nitrile group in aqueous KOH solution was hydrolyzed into carbonyl and amine groups during hydrolysis. The unchanged pyridine-related groups (I) still remained as the majority group. Related amine groups appeared near 399.9 eV (III) against decreased nitrile^24,26^. It is noteworthy that the carbonyl group was formed simultaneously with the amine group. The results of XPS C1s analysis (Table 1) provided evidence for an increase in the oxygen content^27,28^.

**Table 1:** Concentration data for C1s and N1s obtained via hydrolysis, and the change in Pt4f. after carbonisation obtained through XPS analysis.

Species	Binding energy (eV)	Pristine (%)	Hydrolyzed (%)
**C1s**			
sp^3^	284.7	46.0	45.6
sp^3^	286.1	19.8	13.5
C–N/C–O	287.1	23.0	28.0
COO/N–C–O	288.9	7.9	9.4
π–π*	290.8	3.3	3.5
**N1s**			
Nitrile/pyridine	398.9	79.4	59.8
Amide	399.9	–	18.6
Pyridine-N-oxide	400.5	20.6	21.6
**After Pt(acac)**_**2**_** loading pristine and hydrolyzed PAN nanofiber paper**			
**Pt4f**			
PtO	73.0 76.2	86.5	56.8
PtO_2_	74.7 77.9	16.5	43.2
**After carbonation of Pt-loaded PAN nanofiber paper**			
**Pt4f**			
Pt	71.1 74.4	55.9	62.0
PtO	72.2 75.2	44.1	38.0

A similar trend was observed in the Pt4f. peak. The Pt4f. peak of the pristine PAN nanofiber sprayed with Pt(acac)_2_ solution comprised two peaks near 73.0 and 76.2 eV (Fig. 6a). These peaks were assigned to Pt4f7/2 and f5/2 of PtO in Pt(acac)_2_, respectively. Furthermore, in the hydrolyzed nanofiber, two new peaks were found near 74.7 and 77.9 eV, and they were assigned to Pt_4_f7/2 and f5/2 of the Pt atoms respectively, which are interacted with oxygen atoms on the nanofiber surface to form PtO_2_^29^. The areal intensity of these peaks was almost identical to that of the PtO peaks, indicating that the number of active nucleation sites had almost trebled in comparison with that of the pristine samples. The development of nucleation sites was also related to the increase in the specific surface area from 33 to 138 m^2^/g after hydrolysis. The oxide peaks related to PtO and PtO_2_ formation disappeared after carbonization at 800 °C, and consequently, the peak positions shifted to 71.1 and 74.5 eV, which could be identified as peaks of bare Pt atoms (Fig. 6b). The PtO_2_ peaks that appeared after hydrolysis disappeared completely upon carbonization. This is an advantage of hydrolysis, namely, it promotes high catalyst efficiency.

### Electrocatalytic activity for methanol oxidation

To investigate the effect of hydrolysis on the catalytic activity of Pt-loaded PAN-based nanofiber paper, we characterized the electrocatalytic activity of the nanofiber paper for methanol oxidation in an electrolyte of 0.5 M H_2_SO_4_ + 1.0 M CH_3_OH by using cyclic voltammetry (CV) at a scan rate of 20 mV/s. Electrocatalytic activity is directly involved in a direct methanol fuel cell. The CV curve consisted of two main curves: forward oxidation (If) and backward oxidation (Ib) curves. In general, the catalytic process involves several reactions, as follows^30,31^:

1 $$ {\text{CH}}_{3} {\text{OH}} + {\text{H}}_{2} {\text{O}} \to {\text{CO}}_{2} + 6{\text{H}}^{ + } + 6{\text{e}}^{ - } $$

2 $$ {\text{Pt}} + {\text{CH}}_{3} {\text{OH}} \to {\text{Pt}}{-}\left( {{\text{CO}}} \right){\text{ads}} + 4{\text{H}}^{ + } + 4{\text{e}}^{ - } $$

3 $$ {\text{Pt}}{-}\left( {{\text{CO}}} \right){\text{ads}} + {\text{H}}_{2} {\text{O}} \to {\text{Pt}} + {\text{CO}}_{2} + 2{\text{H}}^{ + } + 2{\text{e}}^{ - } $$

4 $$ {\text{C}} - {\text{OH}} + {\text{Pt}}{-}\left( {{\text{CO}}} \right){\text{ads}} \to {\text{C}} + {\text{Pt}} + {\text{CO}}_{2} + {\text{H}}^{ + } + {\text{e}}^{ - } $$

**Figure 7:**
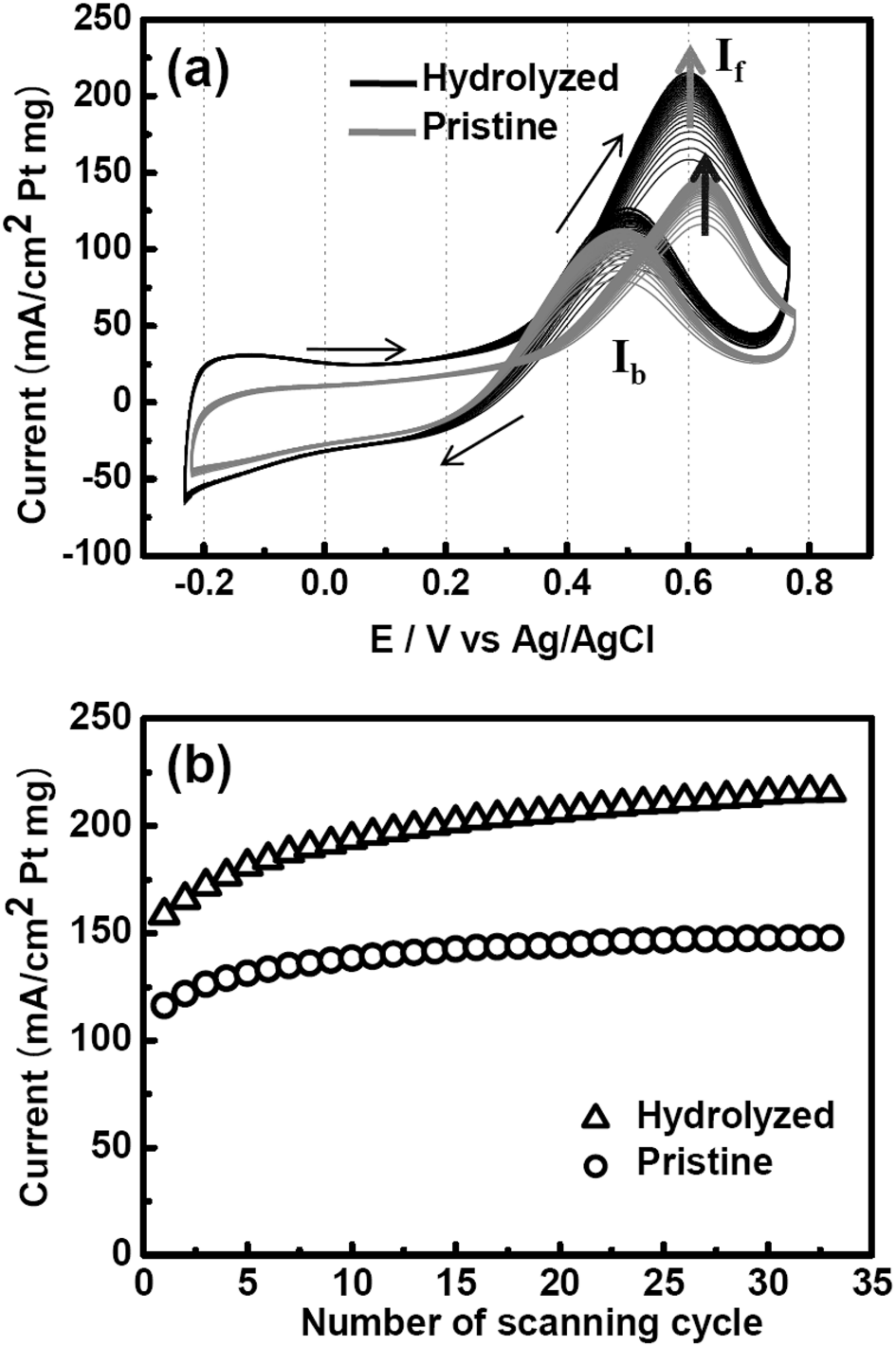
(**a**) Cyclic voltammetry of pristine and hydrolysed PAN-based nanofibers with Pt catalysts. (**b**) Long-term electrocatalytic cycling stability of Pt-loaded PAN-based nanofibers.

The first two equations pertain to the forward oxidation reaction of methanol. The first equation simply shows the complete dissociation of methanol into CO_2_ gas, and the second describes the partial oxidation (poisoning) of Pt atoms by hydroxyl groups. Equation (3) is a backward oxidation reaction for the oxidized Pt atoms, and Eq. (4) could be involved in complete CV scans^30^. The functional groups on the carbon nanofibers such as carboxylic and hydroxyl groups facilitate the formation of Pt–CO ads sites. The ratio of the forward maximum current to the backward current (If/Ib) indicates whether Pt atoms have been completely oxidized without being poisoned, and therefore, it is a good measure of catalytic activity. The If/Ib ratio for Pt catalysts is generally below 1 (it is 0.88 for Pt/CNT and 0.74 for Pt/C)^30,31^. This value was 1.83 for our hydrolyzed sample, much lower than the value (2.30) for Pt_52_Ru_48_/C^28^, but comparable to the value (1.47) for Pt–Co catalysts (Fig. 7a)^31^. This result indicates that our Pt nanoparticles prepared by hydrolysis showed a lower oxidation tendency than the best-known Pt-Ru catalyst. The current density increased with the number of CV cycles (Fig. 7b), indicating the removal of impurities from the surface of the Pt nanoparticles, and the current saturated after 30 cycles. The maximum current reached 213 mA/cm^2^ Pt mg, a value considerably greater than that (50–80 mA/cm^2^ Pt mg) for E-TEK Pt/C^32^. This high current density is attributed to the uniform and monodisperse distribution of Pt nanoparticles over the entire nanofiber.

## Conclusions

In summary, we have demonstrated a loading method for Pt nanoparticles. Electrospun PAN nanofiber paper was used as a substrate and hydrolyzed for increasing the number of nucleation sites for Pt(acac)_2_. The enhancement of Pt adsorption was confirmed through XPS analysis and zeta potential measurement. Uniformly distributed Pt nano particles with sizes of about 3 nm were obtained on the surface of the hydrolyzed nanofibers. The catalytic activity of the Pt-loaded nanofiber paper manifested as a high catalytic current that was nearly thrice the catalytic current for E-TEK Pt/C. The results of this study could be useful for achieving high catalytic activity.
